# Chronic Hyponatremia Following Mild Traumatic Brain Injury: A Case of Persistent Syndrome of Inappropriate Antidiuretic Hormone Secretion (SIADH) in a Young Adult

**DOI:** 10.7759/cureus.114698

**Published:** 2026-08-17

**Authors:** Felicita M Tayong, Amena Hossain Keya, Nida Gul, Wajid Ali, Nouman Khan, Haroon Khan, Samra Miraj

**Affiliations:** 1 Department of Neurosurgery, University of Florida College of Medicine, Gainesville, USA; 2 College of Medicine, University of Science, Arts and Technology, Olveston, MSR; 3 Department of Internal Medicine, Sir Salimullah Medical College, Dhaka, BGD; 4 Department of Medicine, Lady Reading Hospital Peshawar, Peshawar, PAK; 5 Department of Neurological Surgery, Lady Reading Hospital Peshawar, Peshawar, PAK; 6 Department of Emergency Medicine, Lady Reading Hospital Peshawar, Peshawar, PAK; 7 Department of Internal Medicine, Lady Reading Hospital Peshawar, Peshawar, PAK

**Keywords:** hyponatremia, persistent siadh, road traffic accident, syndrome of inappropriate antidiuretic hormone secretion (siadh), traumatic brain injury

## Abstract

Hyponatremia is a well-recognized complication of traumatic brain injury (TBI), most commonly resulting from syndrome of inappropriate antidiuretic hormone secretion (SIADH) or cerebral salt wasting (CSW) syndrome. While SIADH is frequently described following moderate to severe TBI, persistent SIADH after mild TBI is rare and has been infrequently reported.

A 25-year-old man was admitted to Lady Reading Hospital following a road traffic accident resulting in mild TBI. He experienced an uncomplicated neurological recovery, and neuroimaging showed no significant intracranial abnormalities. During hospitalization, he developed symptomatic hyponatremia. Biochemical evaluation, including the assessment of serum and urine osmolality and urinary sodium, was consistent with SIADH after alternative causes of hyponatremia were excluded. Initial management with fluid restriction resulted in the normalization of serum sodium levels. However, recurrent episodes of hyponatremia occurred over the ensuing months, necessitating prolonged fluid restriction and regular outpatient monitoring. No other underlying cause of persistent SIADH was identified despite further evaluation.

This case demonstrates that persistent SIADH can occur even after mild TBI despite normal neuroimaging and an initially favorable neurological recovery. Clinicians should maintain a high index of suspicion for SIADH in patients who develop hyponatremia following mild head injury and recognize the potential for recurrence. Long-term follow-up with the periodic monitoring of serum sodium may facilitate timely intervention and help prevent complications associated with chronic hyponatremia.

## Introduction

Traumatic brain injury (TBI) is a major cause of neurological morbidity and may be associated with a wide spectrum of neuroendocrine complications. Pituitary dysfunction has been increasingly recognized following TBI and can involve both the anterior and posterior pituitary. Chronic hypopituitarism has been reported in 15%-50% of patients, with growth hormone deficiency being the most common endocrine abnormality, followed by deficiencies of adrenocorticotropic hormone, gonadotropins, and thyroid-stimulating hormone [1-3].

In terms of clinical course, acute hyponatremia develops over a short period, whereas recurrent hyponatremia refers to repeated episodes of reduced serum sodium following previous correction. Persistent hyponatremia refers to continued or repeatedly recurring low serum sodium over an extended period rather than resolution during the expected acute post-injury phase [4].

Disorders of sodium homeostasis are among the most common endocrine complications during the acute phase of TBI. Hyponatremia occurs in approximately 20%-50% of patients and is most frequently caused by syndrome of inappropriate antidiuretic hormone secretion (SIADH), although cerebral salt wasting (CSW) syndrome should also be considered in the differential diagnosis [5]. Conversely, central diabetes insipidus due to vasopressin deficiency may result in hypernatremia and has been reported in 3%-26% of patients following TBI [6].

SIADH is characterized by impaired renal water excretion caused by inappropriate antidiuretic hormone (ADH) activity, resulting in hypotonic, usually euvolemic, hyponatremia with inappropriately concentrated urine and elevated urinary sodium. In contrast, cerebral salt wasting (CSW) is characterized by renal sodium loss leading to a reduction in extracellular fluid volume and hypovolemic hyponatremia. Distinguishing these conditions is clinically important because their management differs, with fluid restriction generally used for SIADH and volume and sodium replacement required for CSW [7].

Hyponatremia is associated with significant morbidity and is classified according to serum sodium concentration as mild (130-135 mmol/L), moderate (125-129 mmol/L), or severe (<125 mmol/L) [4]. Acute hyponatremia may present with neurological manifestations such as headache, confusion, seizures, or reduced consciousness, whereas chronic hyponatremia has been associated with gait disturbances, cognitive impairment, falls, osteoporosis, fractures, and prolonged hospitalization [8,9]. In patients with TBI, these manifestations may overlap with the neurological sequelae of brain injury, making prompt recognition particularly challenging.

Although SIADH is well-described after moderate and severe TBI, persistent SIADH following mild TBI has been infrequently reported. Furthermore, the relationship between the severity of head injury and the development of hyponatremia remains uncertain, with conflicting findings across published studies [10]. We present the case of a young man who developed persistent, recurrent SIADH after mild traumatic brain injury despite normal neuroimaging and an initially uncomplicated neurological recovery, highlighting the importance of prolonged biochemical surveillance even in patients with seemingly minor head injuries.

## Case presentation

A 25-year-old man was brought to the emergency department of Lady Reading Hospital Peshawar following a road traffic accident involving a motorcycle collision in June 2024. On presentation, his Glasgow Coma Scale (GCS) score was 13/15 (E3V4M6) with brief disorientation but no focal neurological deficits. He sustained multiple orthopedic injuries, including fractures of the right tibia and left clavicle, along with multiple soft tissue contusions. There was no evidence of cervical spine injury. Baseline renal function was normal, with a serum creatinine level of 0.9 mg/dL.

Within 24 hours of admission, his neurological status improved to a GCS score of 15/15 [11]. A non-contrast computed tomography (CT) scan of the brain showed no acute intracranial hemorrhage, cerebral edema, or other structural abnormalities. Despite this apparent neurological recovery, the patient continued to experience impaired attention, short-term memory difficulties, and intermittent episodes of confusion.

On the fourth day of hospitalization, he developed nausea, generalized weakness, and a mild headache. Laboratory investigations revealed severe hyponatremia with a serum sodium concentration of 118 mmol/L. Further biochemical evaluation demonstrated a low serum osmolality of 245 mOsm/kg, an inappropriately elevated urine osmolality of 890 mOsm/kg, and a urine sodium concentration of 105 mmol/L. Clinical examination demonstrated euvolemia, with no evidence of dehydration or fluid overload. Urine output remained approximately 90-110 mL/hour.

Further evaluation excluded other causes of hyponatremia. Thyroid function tests and early-morning serum cortisol levels were within normal limits, making hypothyroidism and adrenal insufficiency unlikely. There was no history of diuretic use or other medications known to precipitate hyponatremia, apart from routine analgesics and prophylactic antibiotics administered during hospitalization. The absence of clinical evidence of hypovolemia, together with hypotonic hyponatremia, inappropriately concentrated urine, and elevated urine sodium, favored SIADH over cerebral salt wasting. Based on the clinical and biochemical findings, a diagnosis of syndrome of inappropriate antidiuretic hormone secretion (SIADH) secondary to mild traumatic brain injury was established.

The patient was managed conservatively with fluid restriction to approximately 1.0-1.2 L/day. No hypertonic or isotonic saline, oral urea, vasopressin receptor antagonists, or other specific pharmacological therapies were administered. His serum sodium gradually increased to 130 mmol/L over the subsequent 72 hours, with the complete resolution of symptoms. He was discharged with instructions regarding continued fluid restriction and outpatient follow-up.

During the following six months, the patient experienced multiple episodes of recurrent hyponatremia, with serum sodium concentrations ranging from 120 to 125 mmol/L, predominantly following nonadherence to the prescribed fluid restriction. Serum sodium concentrations during these episodes ranged from 120 to 125 mmol/L and were accompanied by nausea, lethargy, impaired concentration, and generalized weakness. Repeat biochemical investigations consistently demonstrated findings compatible with SIADH. Each recurrence responded to stricter fluid restriction, occasionally requiring restriction to 800 mL/day, with the subsequent normalization of serum sodium levels.

At the most recent follow-up, the patient remained neurologically stable without focal deficits but continued to require long-term fluid restriction to maintain normonatremia, consistent with persistent SIADH following mild traumatic brain injury.

The complete laboratory investigations obtained at the time of initial SIADH diagnosis are summarized in Table 1.

**Table 1:** Laboratory investigations at the initial diagnosis of SIADH SIADH, syndrome of inappropriate antidiuretic hormone secretion; TSH, thyroid-stimulating hormone

Analyte	Value	Normal range
Plasma sodium	118 mmol/L	135-145 mmol/L
Plasma potassium	4.3 mmol/L	3.5-5.5 mmol/L
Plasma osmolality	245 mOsm/kg	280-295 mOsm/kg
Urine sodium	105 mmol/L	>30 mmol/L
Urine osmolality	890 mOsm/kg	50-1200 mOsm/kg
Urine output	2160-2640 mL/day	800-2000 mL/day
Plasma glucose	92 mg/dL	70-100 mg/dL
Serum creatinine	0.9 mg/dL	0.7-1.2 mg/dL
TSH	1.8 mIU/L	0.4-4.0 mIU/L
Free T4	1.2 ng/dL	0.8-1.8 ng/dL
Serum cortisol (morning)	18 µg/dL	5-25 µg/dL

Despite repeated clinical and biochemical evaluations during follow-up, no alternative cause of persistent euvolemic hyponatremia was identified. Thyroid and adrenal function remained normal, and there was no clinical or radiological evidence of pulmonary disease, malignancy, or central nervous system infection. The patient continued to require long-term fluid restriction to maintain normal serum sodium levels, with recurrent hyponatremia occurring whenever fluid restriction was not maintained. Although the temporal association between the TBI and the development of SIADH supports TBI as the most likely precipitating factor, a definitive causal relationship cannot be established from a single case.

## Discussion

Syndrome of inappropriate antidiuretic hormone secretion (SIADH) is a heterogeneous disorder characterized by impaired water excretion due to the inappropriate secretion of antidiuretic hormone (ADH) despite normal or low plasma osmolality. Common etiologies include malignancies, particularly small-cell lung carcinoma, central nervous system disorders, pulmonary diseases, and various medications such as antidepressants and proton pump inhibitors [12]. In the present case, repeated clinical and biochemical evaluations excluded these alternative causes, supporting traumatic brain injury (TBI) as the most likely precipitating factor.

The pathophysiology of SIADH following TBI is not completely understood and is likely multifactorial. Proposed mechanisms include injury to the hypothalamus, posterior pituitary, or pituitary stalk, resulting in dysregulated non-osmotic ADH secretion. Damage to hypothalamic osmoreceptors and brainstem cardiovascular regulatory centers may further impair the normal control of water balance [13,14]. In addition, pain, physiological stress, nausea, and inflammatory responses associated with acute trauma may transiently increase ADH release, contributing to the development of hyponatremia during the early post-injury period [4].

Hyponatremia is a well-recognized complication of moderate and severe TBI, with SIADH representing one of its most common causes. However, persistent or recurrent SIADH following mild TBI is uncommon and has been infrequently described in the literature. Our patient developed severe hyponatremia despite normal neuroimaging and rapid neurological recovery. More importantly, he experienced recurrent episodes of hyponatremia over a six-month period that required prolonged fluid restriction. This prolonged clinical course suggests that endocrine dysfunction may persist even when the initial neurological injury appears clinically mild.

The diagnosis of SIADH requires the careful exclusion of other causes of euvolemic hyponatremia. In our patient, clinical examination confirmed euvolemia, while thyroid and adrenal function tests remained normal throughout follow-up. There was no evidence of pulmonary disease, malignancy, central nervous system infection, or medication-induced hyponatremia. The combination of hypotonic hyponatremia, elevated urine osmolality, increased urine sodium concentration, and sustained response to fluid restriction strongly supported the diagnosis of persistent SIADH.

This case highlights an important clinical consideration: the severity of endocrine complications following TBI may not parallel the initial neurological or radiological findings. Patients with apparently mild head injury may still develop significant and prolonged disturbances in sodium homeostasis. The regular monitoring of serum sodium after discharge, particularly in patients with persistent neurological or constitutional symptoms, may facilitate earlier recognition and treatment, thereby reducing the risk of recurrent symptomatic hyponatremia and its associated complications.

## Conclusions

This case illustrates a possible prolonged association between mild traumatic brain injury and recurrent SIADH-like hyponatremia despite normal neuroimaging and an initially favorable neurological recovery. Recurrent symptomatic hyponatremia may require prolonged fluid restriction and monitoring. Clinicians may consider periodic serum sodium monitoring in selected patients with documented or recurrent hyponatremia and persistent symptoms following mild TBI. However, a causal relationship cannot be definitively established from a single case, and further studies are needed to clarify the association between mild TBI and prolonged disturbances of sodium homeostasis.
